# Manganese Phytoremediation Potential of *Koelreuteria paniculata*: Detoxification Mechanisms, Chemical Speciation, and Ultrastructural Adaptations

**DOI:** 10.3390/plants14182867

**Published:** 2025-09-15

**Authors:** Wanyi Zhou, Hao Wang, Huaizhong Jiang, Muhan Zhang, Pufeng Qin, Yonghua Chen

**Affiliations:** 1 College of Environment and Ecology, Hunan Agricultural University, Changsha 410128, China; haizwy@163.com; 2 School of Ecology and Environment, Central South University of Forestry and Technology, Changsha 410004, China; whlikuee@163.com (H.W.);

**Keywords:** manganese slag, woody plants for phytoremediation, mine ecological restoration, heavy metal stress response, subcellular distribution

## Abstract

*Koelreuteria paniculata* demonstrates significant potential for remediating manganese (Mn)-contaminated soils, particularly in mining areas. This study investigated its tolerance and enrichment mechanisms through pot experiments under varying Mn stress (0–15 mmol·L^−1^). The results revealed a typical “low-promotion and high-suppression” response, with optimal growth observed at 5 mmol·L^−1^ Mn. The species exhibited a strong capacity for Mn accumulation, primarily in the roots (up to 2910.24 mg·kg^−1^), though the enrichment factor decreased at higher concentrations. Physiological and subcellular distribution analyses indicated that low Mn levels enhanced chlorophyll content and antioxidant enzyme activities, while excessive stress induced membrane lipid peroxidation. Crucially, tolerance was attributed to effective Mn immobilization in root cell walls (46–76%) and vacuolar compartmentalization in leaves (46–52%), which prevented metal translocation to sensitive organelles. These findings clarify the physiological mechanisms behind Mn tolerance in *K. paniculata* and support its use in practical Mn phytoremediation.

## 1. Introduction

China’s demand for manganese resources is substantial, with a broad range of applications. It is an indispensable raw material for the steel industry and plays a crucial role in the development of new energy, materials, pharmaceuticals, and chemical industries [1], making it of great significance for national development and social progress. China’s manganese resources primarily consist of manganese carbonate, with an average grade of less than 20%. These low-grade ores are the main raw material for the production of electrolytic manganese metal. Electrolytic manganese slag (EMS) contains high levels of soluble Mn^2+^ (5000–10,000 mg·kg^−1^), which can leach into water and soil, causing Mn accumulation beyond natural levels (typically 20–1000 mg·kg^−1^). Excessive Mn (>1500 mg·kg^−1^ in soil) harms ecosystems and human health, necessitating urgent remediation [2,3].

Phytoremediation, as an eco-friendly and sustainable approach, has gained considerable attention for its potential in addressing heavy metal contamination. This technology employs specialized plants to absorb and accumulate heavy metals (HMs) from soil, effectively mitigating environmental pollution while enhancing resource recovery efficiency. This technology is favored due to its environmental friendliness, low economic cost, and long-term benefits. The core of this technology lies in selecting plants that exhibit strong resistance and accumulation capabilities for HMs [4]. Among these, woody plants, due to their large biomass, extensive root systems, and drought tolerance [5], present distinct advantages in the field of phytoremediation. Furthermore, numerous studies have shown that woody plants can affect the HMs in manganese slag through mechanisms such as extraction, volatilization, adsorption, and fixation [6], effectively reducing their bioavailability and mobility [7]. For example, tree species such as *Salix sp*. [8], *Populus L*. [9], *Eucalyptus spp*. [10], and *K. paniculata* [11] have been shown to be efficient in remediating manganese-contaminated sites.

Woody plants can cope with manganese stress through a variety of physiological and biochemical processes to protect themselves from heavy metal toxicity. On one hand, they selectively absorb and accumulate heavy metal ions through their roots [12]. As transpiration occurs, some heavy metal ions are transported upward to the aerial parts of the plant [13]. On the other hand, woody plants stabilize more heavy metal ions through root adsorption to reduce their transport to the aerial parts of the plant, thus protecting the plant from toxicity [7]. Moreover, woody plants can transfer HMs from their roots to their stems and leaves, resulting in a higher concentration of HMs in the aerial parts than in the roots [14], allowing them to sustain growth. During this process, the fixation of HMs in the cell walls and vacuolar compartmentalization can reduce their concentration [14]. Simultaneously, under heavy metal stress, woody plants generate excessive reactive oxygen species (ROS), such as superoxide (O_2_^−^), hydroxyl radicals (OH^−^), and hydrogen peroxide (H_2_O_2_), which affect plant cell metabolism and membrane systems, inhibiting plant growth. To cope with this stress, woody plants initiate antioxidant defense mechanisms to eliminate excessive ROS [15]. Superoxide dismutase (SOD) is distributed in mitochondria, chloroplasts, and cytoplasm, primarily removing oxidative stress-generated O_2_^−^ [16], which is then converted into hydrogen peroxide (H_2_O_2_) and oxygen (O_2_). Antioxidant enzymes, such as peroxidase (POD) and catalase (CAT), catalyze the conversion of H_2_O_2_ to water, thereby alleviating the increase in H_2_O_2_ [17]. Specifically, CAT promotes the breakdown of H_2_O_2_ into molecular oxygen and water, protecting cells from H_2_O_2_ toxicity [18], while POD functions both to eliminate H_2_O_2_ and detoxify phenolic and amine compounds [19]. Raza et al. [20] found that under cadmium stress, the activity of SOD and POD in tolerant plants increased with time, demonstrating that tolerant plants survive heavy metal stress by activating antioxidant mechanisms to reduce the damage caused by the stress.

*K. paniculata* is a deep-rooted deciduous tree belonging to the *Sapindaceae* family. It is resistant to salt, drought, and poor soil conditions, and it has a fast growth rate and well-developed root system, making it highly adaptable to adverse environments [21]. Our research team previously discovered that in mining waste areas with high heavy metal content, *K. paniculata* exhibits high survival rates and vigorous growth, establishing it as a dominant tree species for heavy metal pollution remediation [22]. However, current research on *K. paniculata* primarily focuses on two directions: first, the physiological response characteristics under manganese stress conditions, including photosynthetic efficiency inhibition and dynamic changes in antioxidant enzyme systems (e.g., SOD, CAT); second, ecological adaptability in actual field environments, typically manifested as growth performance and manganese accumulation efficiency assessment in abandoned manganese mining areas of Hunan. It is noteworthy that although slag-contaminated soil has a complex composition, well-controlled laboratory studies based on single-variable manganese stress conditions can effectively eliminate confounding factors, thereby more accurately elucidating the tree’s specific tolerance mechanisms to manganese and its heavy metal accumulation patterns. Thus, further exploration of the manganese stress tolerance and response mechanisms of *K. paniculata* is crucial.

In this study, a pot experiment was conducted to investigate the manganese enrichment characteristics and response mechanisms of *K. paniculata* under different Mn concentrations. By comparing plant physiological-biochemical responses, metal accumulation, heavy metal speciation, and subcellular structure variations in different plant organs, we aim to reveal the mechanism by which *K. paniculata* contributes to phytoremediation in manganese slag and provide theoretical support for ecological restoration in manganese mining areas.

## 2. Results

### 2.1. Growth Status and Heavy Metal Enrichment Characteristics of K. paniculata Under Mn Stress

**Figure 1:**
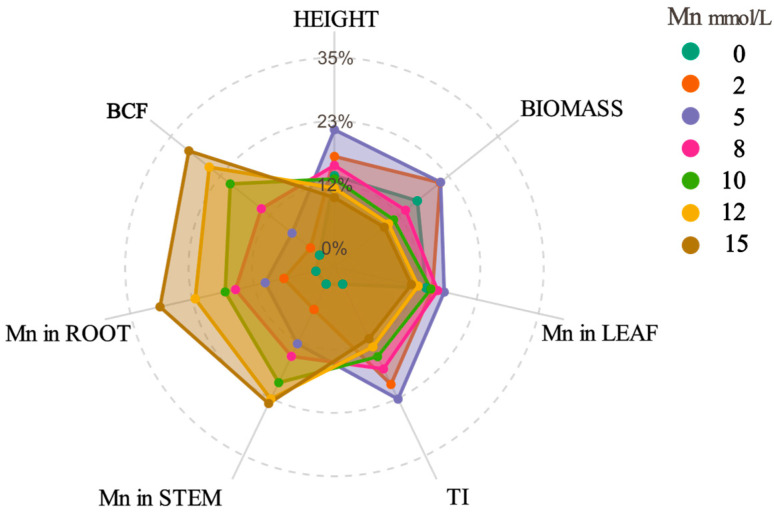
Radar chart showing the relative values of different physiological indices in plants under various manganese (Mn) concentrations (mmol·L^−1^). BCF represents the bioconcentration factor; TI stands for the tolerance index.

The growth status of *K. paniculata* under Mn stress (Table 1 and Figure 1) showed that with increasing Mn stress concentration, plant height, biomass, and root length initially increased and then decreased. At a Mn concentration of 5 mmol·L^−1^, these growth parameters significantly increased (*p <* 0.05), with plant height increasing by 63.66%, biomass by 34.17%, and root length by 24.79%. This indicates that *K. paniculata* exhibits optimal growth under 5 mmol·L^−1^ Mn, which represents its critical Mn tolerance threshold. Below this concentration, plant growth and Mn concentration show a positive correlation, while at higher concentrations, growth is inhibited due to Mn toxicity. TI serves as a key indicator of Mn tolerance, indicating that at Mn concentrations below 8 mmol·L^−1^, TI was above 100%, while at concentrations greater than 10 mmol·L^−^^1^, the plant growth parameters significantly declined (*p <* 0.05). These findings suggest that while *K. paniculata* is tolerant to Mn, its tolerance decreases at higher concentrations.

Mn concentration in the roots, stems, and leaves of *K. paniculata* under Mn stress (Table 2 and Figure 1) showed that, as the Mn concentration increased, the Mn content in each tissue also increased. *K. paniculata* exhibited a higher Mn accumulation in the roots compared to the stems and leaves. The root Mn concentration ranged from 616.71 mg·kg^−1^ to 2910.24 mg·kg^−1^, with stems accumulating between 98.27 mg·kg^−1^ and 430.36 mg·kg^−1^, and leaves between 76.31 mg·kg^−1^ and 371.96 mg·kg^−1^. Notably, the root was the primary organ for Mn accumulation, while the leaf showed the lowest accumulation capacity. BCF decreased as Mn concentration increased, suggesting a reduction in Mn enrichment efficiency with higher concentrations. Specifically, at 2 mmol·L^−1^, the maximum BCF of 0.873 was observed.

### 2.2. Physiological and Biochemical Responses and Structural Equation Modeling (PLS-SEM) of K. paniculata Under Mn Stress

**Figure 2:**
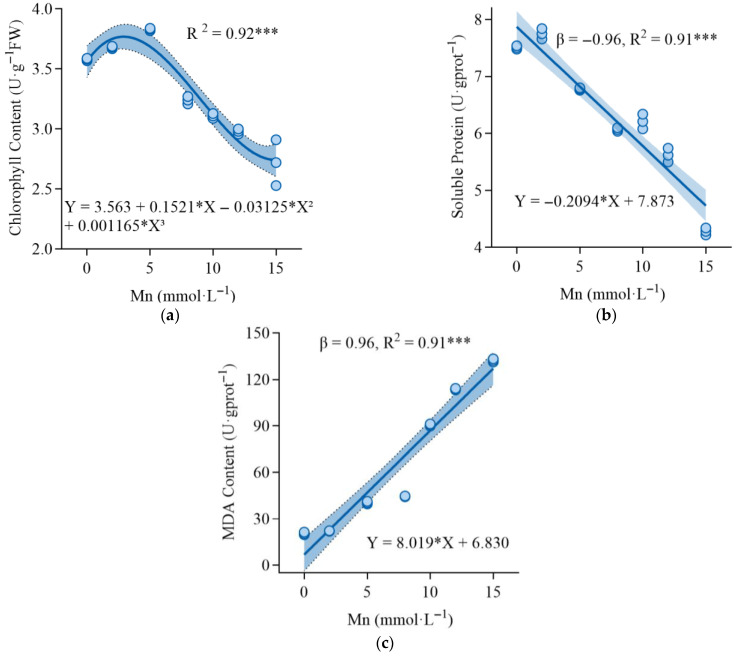
Linear and nonlinear fitting relationships of changes in chlorophyll content (**a**), soluble protein (**b**), and MDA (**c**) levels in *K. paniculata* leaves under manganese stress. *** represents extremely significant difference (*p <* 0.001).

As shown in Figure 2a, the chlorophyll content in the leaves of *K. paniculata* initially increased and then significantly decreased with increasing Mn stress concentration. At a Mn concentration of 5 mmol·L^−1^, the chlorophyll content reached a maximum of 3.83 mg·g^−1^, representing a 6.98% increase compared to the control group (3.58 mg·g^−1^). However, when the Mn concentration increased to 12 mmol·L^−1^, the chlorophyll content significantly decreased to 2.72 mg·g^−1^, reflecting a 28.98% reduction compared to the maximum value. These results indicate that under Mn stress levels below 5 mmol·L^−1^, *K. paniculata* exhibits a positive correlation between growth and Mn concentration, whereas at higher Mn concentrations (above 5 mmol·L^−1^), chlorophyll synthesis is significantly inhibited.

The soluble protein content in *K. paniculata* (Figure 2b) initially increased and then decreased with increasing Mn concentration. At 2 mmol·L^−1^ Mn, soluble protein content peaked at 7.75 mg·g^−1^, but decreased to 4.28 mg·g^−^^1^ at higher concentrations, representing a 43.01% reduction compared to the control. MDA content increased with Mn concentration, reaching 132.40 μmol·g^−1^ at 15 mmol·L^−1^ Mn, a 545.22% increase compared to the control (Figure 2c).

**Figure 3:**
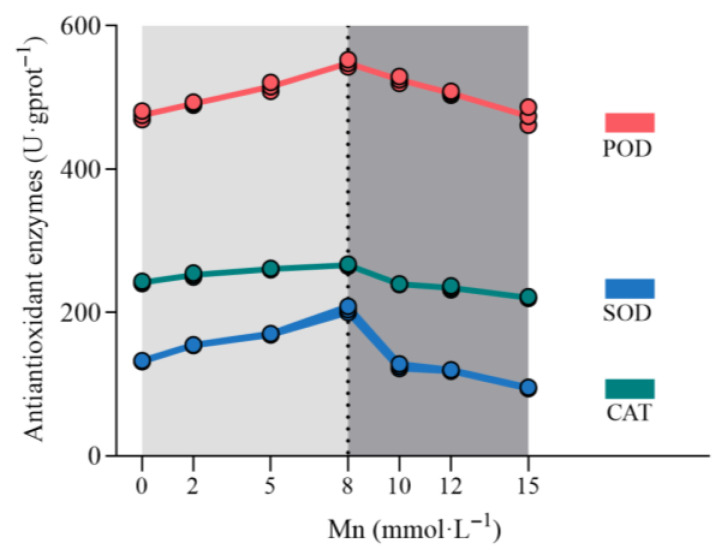
Changes in SOD, POD, and CAT activities in *K. paniculata* under Mn stress (n = 3).

The activities of antioxidant enzymes SOD, POD, and CAT also displayed a trend of initially increasing and then decreasing with increasing Mn stress (Figure 3). The enzyme activities reached their peak at 8 mmol·L^−1^ Mn, showing increases of 54.55%, 15.19%, and 9.98%, respectively, compared to the control. However, when Mn concentration exceeded 8 mmol·L^−1^, the activities of SOD, POD, and CAT declined significantly, with reductions of 28.35%, 0.3%, and 8.74%, respectively, compared to the control. Overall, when the Mn stress concentration was below 8 mmol·L^−1^, the antioxidant enzyme activity in *K. paniculata* increased with rising Mn levels. However, when the stress concentration exceeded 8 mmol·L^−1^, the enzyme activity began to decline with further increases in concentration.

**Figure 4:**
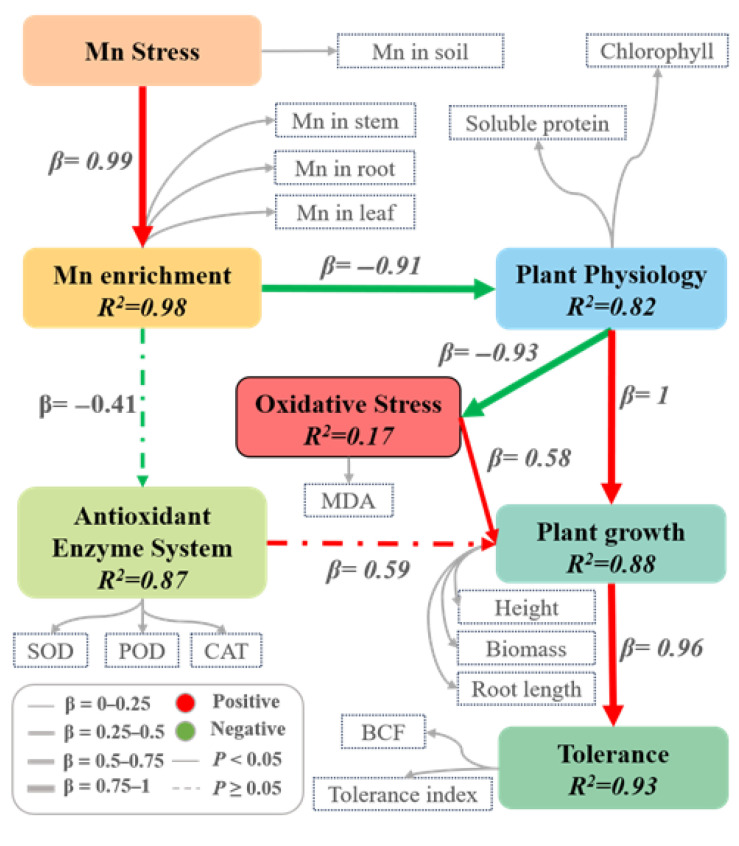
Quantitative PLS-SEM model of adaptive responses in *K. paniculata* across the Mn stress gradient. BCF represents the bioconcentration factor.

We also employed partial least squares structural equation modeling (PLS-SEM) to systematically reveal how manganese (Mn) stress regulates plant growth and tolerance mechanisms in *K. paniculata* (Figure 4). The model demonstrated that Mn stress exerts significant effects primarily through four key mediators: Mn accumulation, plant physiological status, oxidative stress, and antioxidant systems. Mn stress showed an extremely strong positive effect on tissue Mn accumulation (β = 0.99, R^2^ = 0.98), which in turn severely impaired physiological functions (β = −0.91), while dramatically increasing oxidative stress (β = 0.93). The antioxidant system exhibited compensatory regulation of growth (β = 0.59, R^2^ = 0.87), while physiological performance served as a central hub, both mitigating oxidative damage (β = −1.00) and promoting growth (β = 0.58, R^2^ = 0.82). Ultimately, plant growth parameters (height, root length, and biomass) almost completely determined Mn tolerance capacity (β = 0.96, R^2^ = 0.93). These findings collectively establish an integrated framework where Mn stress disrupts growth via accumulation-induced physiological and oxidative damage, while identifying the antioxidant system and physiological homeostasis as critical tolerance determinants in *K. paniculata*.

### 2.3. Subcellular Distribution, Chemical Speciation, and Ultrastructural Alterations in K. Paniculata Under Manganese Stress

**Figure 5:**
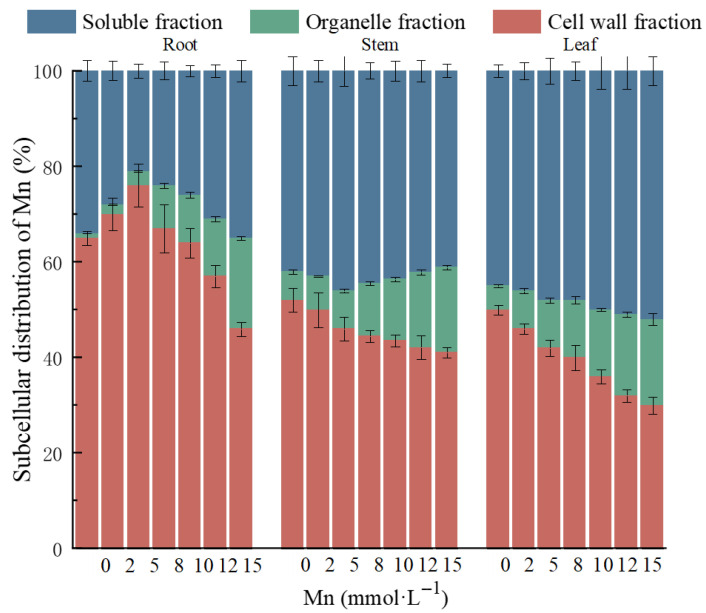
Subcellular distribution of HMs in roots, stems, and leaves of *K. paniculata* under Mn stress.

The subcellular distribution of Mn in the roots, stems, and leaves of *K. paniculata* under Mn stress is shown in Figure 5. With increasing Mn stress, the distribution of Mn in the roots, stems, and leaves was mainly in the cell wall fraction (F1) and soluble fraction (F3), with the least amount in the organelle fraction (F2). Specifically, Mn distribution in the roots was predominantly in the cell wall fraction (46–76%), followed by the soluble fraction (21–35%), and the organelle fraction (2–19%). In the stems and leaves, Mn was more concentrated in the soluble fraction (43–57% in stems, 46–52% in leaves), followed by the cell wall fraction (30–50% in stems, 30–46% in leaves), with the lowest concentration in the organelle fraction (7–13% in stems, 8–18% in leaves). These results indicate that as Mn stress increases, the primary distribution of Mn in *K. paniculata* shifts from the cell wall fraction to the soluble fraction.

**Figure 6:**
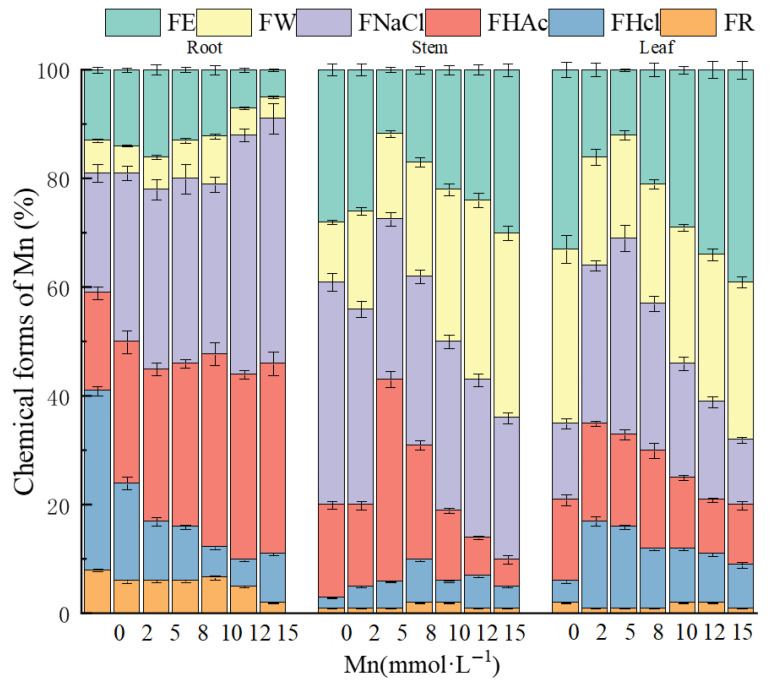
Chemical forms of HMs in the root, stem, and leaf of *K. paniculata* under Mn stress. FE: ethanol-extracted form; FW: water-extracted form; FNaCl: NaCl-extracted form; FHCl: HCl-extracted form; FHAc: HAc-extracted form; FR: residual form.

The chemical form distribution of Mn in the roots, stems, and leaves of *K. paniculata* is shown in Figure 6. In the roots, Mn was predominantly found in NaCl-extracted and HAc-extracted forms, accounting for 25–45% and 15–30%, respectively. These forms were primarily protein-bound or absorbed, as well as in the form of pectate and oxalate salts. In the stems, Mn was mainly found in NaCl-extracted forms (29–39%), followed by deionized water-extracted forms (14–32%), with the Mn occurring primarily as protein-bound or absorbed forms and pectate and water-soluble organic salts. In the leaves, Mn was distributed in ethanol-extracted, deionized water-extracted, and NaCl-extracted forms, with proportions of 12–47%, 18–28%, and 16–28%, respectively. As Mn stress concentration increased, the proportion of NaCl-extracted and HAc-extracted Mn forms increased in the roots, suggesting that the root actively secretes pectate, proteins, and phosphates, which effectively chelate Mn. In the stems, the percentage of NaCl-extracted Mn decreased by 10%, while the deionized water-extracted Mn proportion first decreased and then significantly increased by 18%, indicating that at high Mn concentrations, Mn is primarily transported in water-soluble organic salts. In the leaves, ethanol-extracted and deionized water-extracted Mn increased significantly by 35% and 10%, while NaCl-extracted Mn decreased by 11%, suggesting that at high Mn concentrations, Mn in the leaves transforms into more mobile and toxic forms, such as inorganic salts, amino acid salts, and water-soluble organic salts.

**Figure 7:**
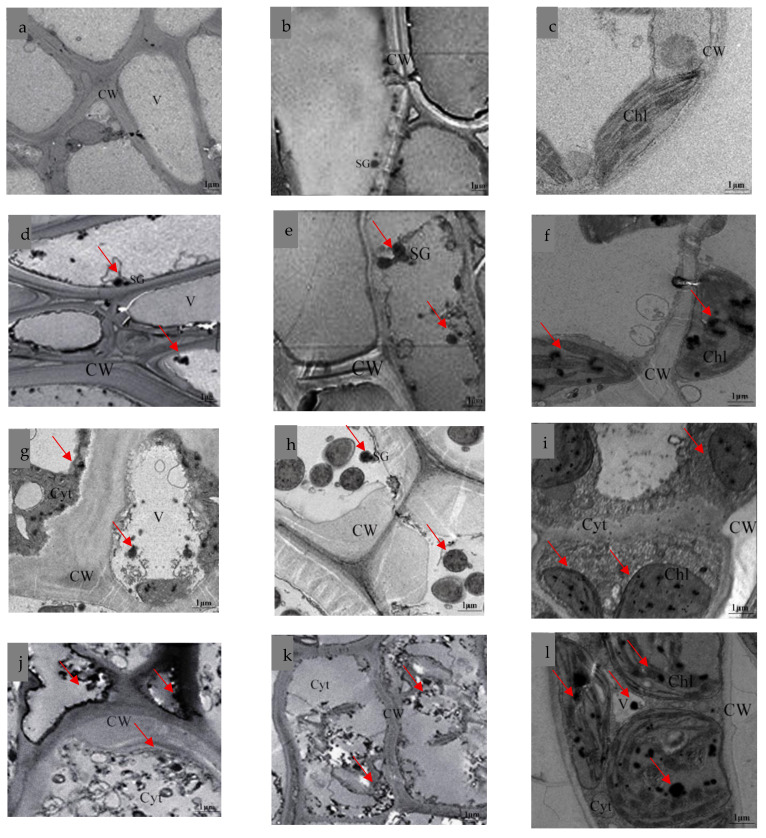
Effects of Mn stress on the microstructure of *K. paniculata* tissues: (**a**–**c**) Microstructure of the root, stem, and leaf cells under control (0 mmol·L^−1^) treatment. (**d**–**f**) Microstructure of the root, stem, and leaf cells under 2 mmol·L^−1^ Mn stress. (**g**–**i**) Microstructure of the root, stem, and leaf cells under 8 mmol·L^−1^ Mn stress. (**j**–**l**) Microstructure of the root, stem, and leaf cells under 15 mmol·L^−1^ Mn stress. CW: cell wall; Cyt: cytoplasm; V: vacuole; Chl: chloroplast; SG: starch grain; The red arrow indicates the area of cellular damage.

Transmission electron microscopy (TEM) revealed significant ultrastructural alterations in the root, stem, and leaf cells of *K. paniculata* under different manganese (Mn) stress levels (0, 2, 8, and 15 mmol·L^−^^1^) (Figure 7). In the control group (0 mmol·L^−^^1^), the cellular structures remained intact, with clearly visible cell walls and no obvious damage to organelles such as vacuoles and chloroplasts. Under 2 mmol·L^−^^1^ Mn treatment, black granular deposits began to appear on the cell walls and chloroplasts. Consistent with previous subcellular distribution data and findings reported by Dou et al. [23], these observations confirm that Mn primarily accumulates in the cell walls and chloroplasts during the early stages of stress. When the Mn concentration increased to 8 mmol·L^−^^1^, a substantial amount of black particles infiltrated the cells, resulting in cell wall rupture, detachment of membrane systems, and organelle damage. This phenomenon aligns with the results of Dou et al. [23], further supporting the notion that excess Mn disrupts cellular compartmentalization. Under 15 mmol·L^−^^1^ Mn stress, severe deformation of cell walls, distortion of membrane structures, vacuole shrinkage leading to plasmolysis, and complete disruption of chloroplasts were observed. These results demonstrate that high concentrations of Mn not only alter the subcellular distribution of Mn but also cause irreversible damage to the cellular ultrastructure, with the extent of damage closely correlated to both the localization and concentration of Mn accumulation.

## 3. Discussion

Manganese hyperaccumulators are typically defined as plants containing more than 10,000 mg·kg^−1^ of manganese in their tissues. Although the maximum manganese content observed in the roots of *K. paniculata* in this study was 2910.24 mg·kg^−1^, it should be noted that these data were obtained from saplings. It is worth emphasizing that most currently reported hyperaccumulator species are herbaceous, whereas woody plants—though often exhibiting lower manganese concentration per unit tissue—possess a large biomass, enabling substantial total manganese uptake. This trait offers significant advantages in remediation applications. More importantly, our recent research revealed that the remediation mechanism of *K. paniculata* extends beyond phytoextraction: it also demonstrates a notable immobilization effect on heavy metals such as manganese, lead, and zinc in manganese tailings, significantly reducing their bioavailability and mobility [22]. This study systematically analyzed the growth indicators of *K. paniculata* under Mn stress, revealing that it possesses manganese tolerance. As Mn stress increased, the plant’s height, biomass, and root length initially increased and then decreased, with a tolerance threshold of 8 mmol·L^−1^ Mn. Below this threshold, TI increased by 23%, 41%, and 4%, respectively, while at concentrations above this threshold, TI decreased by 11%, 23%, and 33%, indicating that high Mn concentrations inhibit growth and metabolism. The root, stem, and leaf tissues showed increasing Mn accumulation with higher Mn concentrations, demonstrating that *K. paniculata* has a strong ability to accumulate Mn, with the root being the main organ for Mn fixation. However, at higher Mn concentrations (≥8 mmol·L^−1^), BCF decreased, suggesting that the plant’s ability to accumulate and fix Mn becomes inhibited. Mn is an essential micronutrient for plant growth and development, involved in multiple key physiological and biochemical processes such as photosynthesis, antioxidant enzyme activation, and nitrogen metabolism. Under low-concentration conditions, manganese acts as a cofactor for various enzymes (e.g., manganese superoxide dismutase and dehydrogenases), promoting cellular metabolism and energy synthesis, thereby significantly enhancing plant growth vigor [24]. This mechanism clearly explains the improved growth indicators—such as plant height, biomass, and root length—observed in *K. paniculata* under low-concentration manganese treatment in this study. Similar findings were reported by Zhang et al. in their investigation of manganese remediation using *Paulownia fortune* [25], further confirming the growth-promoting effects of manganese within an appropriate concentration range on woody plants. Thus, manganese is not only an essential trace element but also plays a critical role in maintaining normal metabolism and stress resistance through its homeostatic regulation in plants.

This study explored the physiological and biochemical responses of *K. paniculata* under varying Mn stress concentrations. The experimental results indicated that chlorophyll content, soluble protein, MDA content, and antioxidant enzyme activities in the leaves of *K. paniculata* were all affected by Mn stress. Specifically, chlorophyll content increased under low Mn stress and decreased under high Mn stress, which is consistent with the changes in chlorophyll content observed in *Dalbergia odorifera* seedlings [26] and *Tamarix aphylla* leaves under Mn stress [27]. The trend in soluble protein content mirrored that of chlorophyll, suggesting that under low Mn stress, *K. paniculata* adapts by secreting soluble proteins to regulate intracellular osmotic pressure. However, at high Mn concentrations, the plant’s defense capacity is overwhelmed, leading to a reduction in soluble protein content. The continuous increase in MDA content reflects intensified membrane lipid peroxidation caused by Mn stress. The role of SOD in the antioxidant system of *K. paniculata* is to remove excess reactive oxygen species (ROS) and convert them into hydrogen peroxide, which is then reduced by POD and CAT into water molecules [16]. The generation of ROS activates the plant’s antioxidant system, causing the activities of SOD, CAT, and POD to initially increase and then decrease [14,16]. Moreover, under high Mn stress, antioxidant enzyme activities significantly decreased, indicating cellular damage due to toxicity. These findings further demonstrate the “low promotion, high suppression” response mechanism of *K. paniculata* to Mn stress. In conclusion, *K. paniculata* adapts to Mn stress by regulating chlorophyll content, soluble protein content, and antioxidant enzyme activities, showing its physiological response mechanism to heavy metal stress.

The cell wall is a critical defense line for fixing HMs and resisting heavy metal stress. When the concentration of HMs exceeds the plant’s tolerance threshold, oxidative stress reactions and cellular damage occur, manifesting as lipid peroxidation [25]. This study reveals the distribution characteristics of Mn in *K. paniculata* cells, showing that Mn is primarily concentrated in the cell wall and soluble fractions, indicating that the root cell wall and leaf vacuoles are the main sites for Mn fixation and sequestration. As Mn stress concentration increases, the Mn content in the cell wall decreases, and free Mn ions gradually accumulate in the cytoplasm, organelles, and vacuoles, suggesting that under high Mn stress, Mn ions can penetrate the cell wall and enter the cell, causing damage to organelles.

Mn exists in various forms in plant tissues, primarily in chelated and complexed states, and different Mn forms exhibit varying degrees of toxicity. Specifically, Mn in inorganic acid salts, amino acid salts, and water-soluble organic acid salts has higher bioactivity and toxicity, followed by pectate and protein-bound forms, and phosphate-bound states, while oxalate-bound and residual forms are the most stable and least mobile [28]. This study shows that in the roots of *K. paniculata*, Mn mainly exists in pectate, protein-bound, and phosphate-bound forms, suggesting that the plant can bind Mn through the secretion of pectate, specific proteins, and phosphates to resist Mn-induced cellular damage. Moreover, the plant adsorbs and fixes Mn in the roots in weakly toxic and less mobile forms to minimize the upward transport of Mn ions. In the stems and leaves, Mn shifts to more toxic and bioavailable forms. As the Mn stress gradient increases, the percentage of water-soluble organic acid-bound Mn in the stem and inorganic acid, amino acid-bound Mn in the leaves rises, indicating that under high Mn stress, *K. paniculata* may transport Mn upward to avoid cellular damage. However, due to decreased Mn fixation ability in the leaves, the bioactivity and toxicity of Mn remain high in the plant. Therefore, the manganese tolerance mechanism of *K. paniculata* involves converting highly active, mobile Mn forms into less toxic and mobile bound forms. This finding is consistent with the conclusions of Cai et al. [29].

Through TEM, we observed that under high Mn stress, the damage to the roots of *K. paniculata* primarily manifested as cell wall rupture. In the stems, the main effects were cell membrane distortion and plasmolysis, while in the leaves, the damage was mainly to organelles, with chloroplast disintegration leading to loss of function. Additionally, large black metal particles were visible in all tissues of *K. paniculata*, likely representing heavy metal precipitation [25]. Under low Mn stress, Mn particles mainly adhered to the cell wall. However, under moderate to high Mn stress, Mn particles infiltrated the vacuoles, cytoplasm, and organelles. These observations further support the conclusion that at high Mn stress, the role of the cell wall in retaining Mn weakens, allowing Mn ions to flow into organelles, causing damage to cellular structures.

## 4. Materials and Methods

### 4.1. Experimental Materials

*K. paniculata* seedlings were purchased from a nursery, with approximately similar biomass and plant height, and their apical buds were removed to standardize the starting plant material. The species identity was confirmed by a professor of botany at our institution. River sand, used for cultivation, was purchased from a construction material store.

### 4.2. Experimental Design

The experiment was conducted in the nursery greenhouse of Central South University of Forestry and Technology, Changsha, Hunan Province, China (28.1789° N, 112.9438° E, 85 m). The environmental conditions inside the greenhouse were controlled as follows: air relative humidity was maintained between 60% and 75%, and the temperature was kept within the range of 25–30 °C. Healthy seedlings of *K*. *paniculata* were selected, and the experiment involved seven Mn stress concentration gradients, with three replicates per gradient. River sand was sterilized with 2% nitric acid and used as the growth medium, placed in plastic pots (29 cm in diameter and 17.8 cm in height), each containing approximately 5 kg of sand. The pH of the sand was adjusted using dilute HCl and NaOH. A modified 1/2 Hoagland nutrient solution was used, where solutions A, B, and C were prepared separately in 1 L bottles, mixed with deionized water, and used in the following proportions: 10 mL of each stock solution per liter of working solution (Tables S1 and S2). Nutrient solution was applied every three days, and after two weeks of natural rain protection in outdoor conditions, MnSO_4_·H_2_O solutions at concentrations of 0, 2, 5, 8, 10, 12, and 15 mmol·L^−1^ were added to each pot (500 mL per application). This concentration range was selected based on our ongoing research in phytoremediation, wherein preliminary experiments indicated that *K. paniculata* fails to survive at Mn concentrations ≥16 mmol·L^−1^, leading to rapid mortality after planting. The experiment was conducted for 37 days, with a total of 10 applications. Regular plant care was performed, including weeding, soil aeration, and periodic shifting of the pots to eliminate location effects. At the end of the experimental period, plant and soil samples were collected. *K. paniculata* was divided into roots, stems, and leaves, with part of the tissue used for fresh weight measurements and another part dried and ground for further analyses.

### 4.3. Measurement Indices and Methods

#### 4.3.1. Plant Growth Indices

Plant height was measured with a tape measure. Plants were carefully excavated using a small hoe to maintain root integrity, transported to the laboratory, and gently rinsed. The roots were then immersed in 5 mmol·L^−1^ Ca(NO_3_)_2_ solution for 15 min, followed by washing with ultrapure water. Residual moisture was blotted with filter paper. Each plant was separated into roots, stems, and leaves. Root growth was analyzed immediately after sampling using a root scanner (WinRHIZO STD4800, Québec, QC, Canada) and associated software (WinRHIZO PRO 2013, Québec, QC, Canada). Fresh plant tissues were rinsed with distilled water and cut into small pieces (2 mm × 2 mm × 2 mm) using a scalpel. The samples were subsequently fixed in prechilled (4 °C) 2.5% glutaraldehyde solution. Ultrastructural observations of various tissues in *K. paniculata* were carried out using a transmission electron microscope (TEM; FEI Tecnai G2 Spirit, Thermo Fisher Scientific, Waltham, MA, USA) at Shiyanjia Lab (www.shiyanjia.com). Plant parts were dried at 105 °C for 30 min and then at 75 °C until they reached a constant weight, after which dry biomass was measured.

#### 4.3.2. Chlorophyll Content, Soluble Protein, and Enzyme Activity

Chlorophyll content was determined using a UV-5100 ultraviolet–visible spectrophotometer (Shanghai Yuanxi Instrument Co., Ltd., Shanghai, China) [30]. Mature leaves from the same nodal position (the fourth functional leaf) were collected from both treated and control groups, with 0.5 g samples weighed, rinsed, and gently blotted dry. Each sample was homogenized in 10 mL of prechilled phosphate buffer (pH 7.8, added in two aliquots) using liquid nitrogen (or an ice bath) for extraction. The homogenate was then centrifuged at 10,500 rpm for 15 min at 4 °C. The resulting supernatant, designated as the enzyme extract, was stored at 4 °C and could be subsequently used for the determination of antioxidant enzyme activities, soluble protein content, and malondialdehyde (MDA) levels. All grinding procedures were performed under low-temperature and light-protected conditions. Total superoxide dismutase (SOD) activity was measured using the NBT [31] method (Sharma & Shukla, 2021); catalase (CAT) activity was measured by ultraviolet absorption [32]; peroxidase (POD) activity was assessed using a standard method [33]; the MDA content was determined using the thiobarbituric acid (TBA) method, while the soluble protein content was measured with the Coomassie Brilliant Blue G-250 colorimetric method [34].

#### 4.3.3. Mn Content in Plants

The collected tissues were dried at 105 °C for 30 min and further dried to constant weight at 75 °C. Dried and weighed plant samples were ground using a mill, sieved, and stored in sealed bags. Precisely 1.000 g of powder from roots, stems, and leaves was weighed into separate 50 mL conical flasks and digested using a wet nitric–perchloric acid digestion method. The flasks were placed on a hotplate, and 10 mL of nitric acid was added to each, followed by the placement of a curved-neck funnel and overnight standing. The following day, the samples were heated on the hotplate. When brown fumes emerged during high-temperature digestion, 3 mL of perchloric acid was promptly added, and the temperature was further increased. Heating was continued until the solution became clear and colorless. After cooling, the digestate was rinsed and diluted to 50 mL with ultrapure water in a volumetric flask. The heavy metal content in each plant part was determined by flame atomic absorption spectrophotometry (FAAS, AA-7002, Thermo Fisher Scientific, Waltham, MA, USA). The bioconcentration factor (BCF) was calculated as the ratio of Mn concentration in the stem and leaves to the Mn concentration in the treatment solution, reflecting Mn accumulation efficiency in *K. paniculata* [35]. The tolerance index (TI) was derived from the relative plant height, biomass, and root length in treated versus control groups, quantifying Mn tolerance across different concentrations [36].

The subcellular distribution of Mn in the roots, stems, and leaves was determined by differential centrifugation [37]. Fresh plant tissues were rinsed with distilled water and homogenized in a prechilled (4 °C) extraction buffer (250 mM sucrose, 1.0 mM dithioerythritol, and 50 mM Tris-HCl, pH 7.5) using quartz sand and a grinding pestle. The homogenate was transferred to centrifuge tubes and centrifuged at 2000 g for 1 min. The resulting pellet was designated as the “cell wall fraction”. The supernatant was further centrifuged at 10,000× *g* for 30 min, yielding a precipitate termed the “organelle fraction”, with the final supernatant referred to as the “soluble fraction”. All procedures were conducted at 4 °C. After digestion with nitric–perchloric acid on a hot plate, Mn concentrations were measured using flame atomic absorption spectrophotometry [38].

Mn content in plant tissues was sequentially extracted using specific chemical extractants to determine different chemical forms of Mn [23], and the concentrations were measured by flame atomic absorption spectrophotometry [38]. The following extractants were applied sequentially for continuous fractional extraction: (1) 80% ethanol; (2) deionized water; (3) 1 M NaCl; (4) 2% acetic acid (HAc); and (5) 0.6 M HCl. The residue remaining after extraction was regarded as the residual fraction. Additional fresh plant tissue was washed with distilled water, homogenized in extractant at a solid-to-liquid ratio of 1:10 (*w*/*v*) with quartz sand and a grinding pestle, and then shaken at 25 °C for 22 h. The homogenate was centrifuged at 5000× *g* for 10 min, and the supernatant was collected, filtered, and stored for further analysis.

### 4.4. Data Analysis Methods

Data were recorded and processed using Microsoft Office Excel 2021. Statistical analyses, including one-way analysis of variance (ANOVA) and Duncan’s multiple range test (*p* < 0.05), were performed using IBM SPSS Statistics (v27.0) to assess differences in plant chlorophyll content, enzyme activities, and heavy metal concentrations. Multivariate relationships between Mn concentrations and plant physiological responses were visualized using a radar chart generated in R (v4.4.3) with the ggplot2 (v3.5.2) package for core visualization, supported by dplyr (v1.1.4.9000) and tidyr (v1.3.1) for data wrangling, and plotthis (v1.1.0) for polar coordinate transformation. To examine the hypothesized structural pathways, we employed partial least squares structural equation modeling (PLS-SEM) using the seminr package (v2.3.4) in R. All data are presented as mean ± standard deviation (SD) from three independent biological replicates (n = 3). Final figures were prepared using Origin 2021, GraphPad Prism (v10.4), and Microsoft Office PowerPoint 2021.

## 5. Conclusions

*K. paniculata* exhibits a remarkable concentration-dependent tolerance to manganese (Mn) stress, with low concentrations promoting growth and high concentrations causing inhibition. This study elucidates three key mechanisms underlying its Mn tolerance: first, Mn is immobilized in root cell walls and vacuoles to limit translocation to aerial parts; second, the maintenance of antioxidant enzyme systems and physiological homeostasis alleviates oxidative damage; and third, Mn predominantly exists in low-activity forms in roots while converting to highly active forms in shoots. These findings indicate that *K. paniculata* employs a cascade mechanism of “root adsorption–stem transport–vacuolar compartmentalization” to cope with Mn stress. The results provide physiological insights supporting the use of *K. paniculata* in the phytoremediation of Mn-contaminated soils.

## Figures and Tables

**Table 1:** Growth status of *K. paniculata* under Mn stress.

Mn Concentration (mmol·L^−1^)	Plant Height (cm)	Biomass (g)	Root Length (cm)	TI (%)
0	22.32 ± 1.89 d	11.12 ± 0.03 b	116.35 ± 1.24 d	/
2	28.27 ± 1.14 b	14.84 ± 1.14 a	126.60 ± 9.13 c	123
5	36.53 ± 1.99 a	14.92 ± 0.13 a	145.19 ± 10.76 a	141
8	25.52 ± 0.98 c	9.20 ± 0.15 c	134.61 ± 5.76 b	104
10	21.34 ± 1.14 d	7.25 ± 0.12 d	124.03 ± 1.95 c	89
12	18.49 ± 1.03 e	6.42 ± 0.06 e	103.67 ± 1.44 e	77
15	15.55 ± 0.49 f	5.71 ± 0.14 f	92.92 ± 4.64 f	67

Data are presented as mean ± standard deviation of three replicates. Different letters in each column indicate significant differences at *p* < 0.05; TI stands for the tolerance index.

**Table 2:** Mn concentration in *K. paniculata* tissues under Mn stress.

Mn Concentration (mmol·L^−1^)	Distribution of Manganese in Different Organs (mg·kg^−1^)			BCF
	Root	Stem	Leaf	
2	616.71 ± 70.15 f	98.27 ± 3.87 f	76.31 ± 1.35 f	0.873 ± 0.03 a
5	1453.55 ± 29.61 e	156.23 ± 2.70 e	167.10 ± 3.88 e	0.647 ± 0.01 b
8	1760.28 ± 25.77 d	247.58 ± 24.01 d	229.81 ± 1.42 d	0.597 ± 0.03 d
10	2401.27 ± 27.70 c	279.45 ± 32.58 c	309.39 ± 5.39 c	0.589 ± 0.04 ef
12	2803.18 ± 58.62 b	372.02 ± 27.43 b	361.28 ± 1.25 b	0.611 ± 0.02 c
15	2910.24 ± 62.79 a	480.36 ± 29.81 a	371.96 ± 22.35 a	0.568 ± 0.03 g

Data are presented as mean ± standard deviation of three replicates. Different letters in each column indicate significant differences at *p* < 0.05; BCF represents the bioconcentration factor.

## Data Availability

All additional datasets supporting the findings of this study are included within the article and Supplementary Materials.

## Supplementary Materials

The following supporting information can be downloaded at: https://www.mdpi.com/article/10.3390/plants14182867/s1.
