# Supplementary material for: Manganese Phytoremediation Potential of Koelreuteria paniculata: Detoxification Mechanisms, Chemical Speciation, and Ultrastructural Adaptations
Source: Plants (Basel). 2025 Sep 15;14(18):2867. doi: 10.3390/plants14182867 (PMC12473184; doi:10.3390/plants14182867)
Supplement: Supplementary file 1 [file plants-14-02867-s001.zip › plants-3835624-supplementary.pdf]

Table S1. Formulation for modified 1/2 Hoagland nutrient solution (100x stock concentrations).

Stock Bottle	Compound Name	Chemical Formula	Amount to prepare 1L of Stock (g)	Main Elements Supplied
Stock A	Calcium Nitrate	$Ca(NO_3)_2{\cdot}4H_2O$	94.20 g	Ca, N
Stock B	Potassium Nitrate	KNO_3	60.60 g	K, N
Stock B	Magnesium Sulfate	$MgSO_4{\cdot}7H_2O$	49.30 g	Mg, S
Stock B	Monopotassium Phosphate	KH_2PO_4	13.60 g	P, K
Stock C	EDTA Ferric Sodium Salt (Fe-EDTA)	NaFe-EDTA	3.67 g	Fe
Stock C	Boric Acid	H_3BO_3	0.286 g	B
Stock C	Manganese Chloride	$MnCl_2{\cdot}4H_2O$	0.181 g	Mn
Stock C	Zinc Sulfate	$ZnSO_4{\cdot}7H_2O$	0.022 g	Zn
Stock C	Copper Sulfate	$CuSO_4{\cdot}5H_2O$	0.008 g	Cu
Stock C	Sodium Molybdate	$Na_2MoO_4{\cdot}2H_2O$	0.0024 g	Mo

[1] Prepare Stock A, B, and C separately. For each bottle, first dissolve the assigned compounds in approximately 800 mL of distilled water with stirring until fully dissolved. Then bring the final volume to 1 L by adding distilled water and mix thoroughly. Store all stocks in amber bottles or a dark location. Note that Stock A is highly hygroscopic and must be tightly sealed after preparation.

Table S2. Elemental concentrations in the final working solution

Nutrient Element	Concentration (mM)	Concentration (mg/L)	Stock Source
Nitrogen (N)	7.50	105.01	Stock A, B
Phosphorus (P)	0.50	15.50	Stock B
Potassium (K)	3.00	117.30	Stock A, B
Calcium (Ca)	2.00	80.16	Stock A
Magnesium (Mg)	1.00	24.31	Stock B
Sulfur (S)	1.00	32.06	Stock B
Iron (Fe)	0.05	2.79	Stock C
Boron (B)	0.023	0.25	Stock C
Manganese (Mn)	0.0045	0.25	Stock C
Zinc (Zn)	0.00038	0.025	Stock C
Copper (Cu)	0.00016	0.01	Stock C
Molybdenum (Mo)	0.00005	0.005	Stock C

[1] To prepare 1 liter of final nutrient solution, combine 10 mL each of Stocks A, B, and C into approximately 800 mL of distilled water while stirring vigorously, rinse measuring devices between additions, bring the total volume to 1 L, mix thoroughly, and adjust the pH to 5.8–6.0 using dilute HCl or NaOH.
